# Assessment of core capacities for the International Health Regulations (IHR[2005]) – Uganda, 2009

**DOI:** 10.1186/1471-2458-10-S1-S9

**Published:** 2010-12-03

**Authors:** Joseph F Wamala, Charles Okot, Issa Makumbi, Nasan Natseri, Annet Kisakye, Miriam Nanyunja, Barnabas Bakamutumaho, Julius J Lutwama, Rajesh Sreedharan, Jun Xing, Peter Gaturuku, Thomas Aisu, Fernando Da Silveira, Stella Chungong

**Affiliations:** 1 Ministry of Health, P.O. Box 7272, Kampala, Uganda; 2 World Health Organisation Country Office, Plot 60, Prince Charles Drive, P.O. Box 24578, Kampala; 3 World Health Organisation - Regional Office for Africa, Cité du Djoué, P.O. Box 06, Brazzaville, Republic of Congo; 4 World Health Organisation Headquarters - Avenue Appia 20; 1211 Geneva 27, Switzerland

## Abstract

**Background:**

Uganda is currently implementing the International Health Regulations (IHR[2005]) within the context of Integrated Disease Surveillance and Response (IDSR). The IHR(2005) require countries to assess the ability of their national structures, capacities, and resources to meet the minimum requirements for surveillance and response. This report describes the results of the assessment undertaken in Uganda.

**Methods:**

We conducted a descriptive cross-sectional assessment using the protocol developed by the World Health Organisation (WHO). The data collection tools were adapted locally and administered to a convenience sample of HR(2005) stakeholders, and frequency analyses were performed.

**Results:**

Ugandan national laws relevant to the IHR(2005) existed, but they did not adequately support the full implementation of the IHR(2005). Correspondingly, there was a designated IHR National Focal Point (NFP), but surveillance activities and operational communications were limited to the health sector. All the districts (13/13) had designated disease surveillance offices, most had IDSR technical guidelines (92%, or 12/13), and all (13/13) had case definitions for infectious and zoonotic diseases surveillance. Surveillance guidelines were available at 57% (35/61) of the health facilities, while case definitions were available at 66% (40/61) of the health facilities. The priority diseases list, surveillance guidelines, case definitions and reporting tools were based on the IDSR strategy and hence lacked information on the IHR(2005). The rapid response teams at national and district levels lacked food safety, chemical and radio-nuclear experts. Similarly, there were no guidelines on the outbreak response to food, chemical and radio-nuclear hazards. Comprehensive preparedness plans incorporating IHR(2005) were lacking at national and district levels. A national laboratory policy existed and the strategic plan was being drafted. However, there were critical gaps hampering the efficient functioning of the national laboratory network. Finally, the points of entry for IHR(2005) implementation had not been designated.

**Conclusions:**

The assessment highlighted critical gaps to guide the IHR(2005) planning process. The IHR(2005) action plan should therefore be developed to foster national and international public health security.

## Background

The International Health Regulations (IHR[2005]) are a set of legally binding regulations for all World Health Organisation (WHO) Member States. They aim to harmonise the protection of public health while avoiding unnecessary disruption of trade and travel through the development of effective global alert, surveillance and response strategies for all priority public health events.

The IHR(1969) were used by WHO Member States to guide international prevention and control of infectious diseases until June 2007. The IHR(1969) obliged WHO Member States to notify the WHO of cholera, plague and yellow fever outbreaks in their territories. In addition, the IHR(1969) included requirements for health and vaccination certificates for travellers from infected to non-infected areas; deratting, disinfecting and disinsecting of ships and aircraft; as well as detailed health measures at airports and seaports in the territories of WHO Member States [1].

Rapid globalisation and the emergence of new diseases and hazards in the 21^st^ century rendered the IHR(1969) inadequate to deal with the increased risk of international spread of public health risks and hazards. Contagious illnesses spread farther and faster than ever with the increase in plane traffic. New outbreaks of Ebola in Zaire [2] and Severe Acute Respiratory Syndrome in China [3] and Marburg [4] emerged with implications for international travel and trade and required coordinated international response. The IHR(1969) also wholly depended on the affected country to make an official notification to the WHO once cases were diagnosed and lacked mechanisms to foster collaboration between the WHO and a country in which public health events with potential for international spread were occurring. The IHR(1969) also lacked effective incentives to encourage compliance by Member States. The limitations of the IHR(1969) therefore paved way for its revision to address the above-mentioned gaps and provide real-time information to inform formulation of measures to prevent international disease spread. The revised IHR(2005) was adopted by the 58^th^ World Health Assembly on 23 May 2005 and entered into force on 15 June 2007 [5].

Uganda is using the WHO Africa Region Integrated Disease Surveillance and Response (IDSR) Strategy for the control of communicable diseases [6]. The strategy promotes the integration of surveillance activities for priority conditions, taking advantage of common surveillance and support functions at all levels. Uganda adopted the strategy in 2000 [7] and it is being used as the vehicle for implementing the IHR(2005) [8]. Article 5 and Annex 1a of the IHR(2005) require countries to assess the ability of existing national structures, capacities, and resources to meet the minimum requirements for surveillance and response, within two years following the entry into force of these regulations. This assessment is meant to inform the process of developing and implementing action plans to ensure that all the core capacity requirements for the IHR(2005) are established and maintained throughout the country. In line with this requirement, the Ministry of Health (MoH) Uganda, with support from the WHO, conducted an assessment of the core capacities required for implementation of the IHR(2005) during the period 12 to 13 October 2009. The purpose of the assessment was to obtain baseline information on the current status of IHR(2005) core capacities for all the five hazards and at points of entry so as to facilitate the development of an action plan to guide the establishment and maintenance of the capacities in the country.

Uganda is located in East Africa and is bordered by Kenya to the East; Sudan to the North; Democratic Republic of Congo to the West; Tanzania to the South; and Rwanda to the Southwest. It has a population of 30 million people with an area of 241,038 square kilometres, of which the land area covers 197,323 square kilometres. There are four physical regions, namely: Central, Western, Northern and Eastern. The regions are divided into districts and at the time of the assessment, there were 80 districts in the country.

## Methods

### Preparation

We conducted a descriptive cross-sectional assessment using the protocol developed by the WHO for assessing national surveillance and response capacities for the IHR(2005) [9]. The assessment was coordinated by the Ugandan National IHR Focal Point (NFP) with technical and logistical support from the WHO. The assessment team included IHR(2005) stakeholders from national and district levels. A two-day pre-assessment workshop was held to examine the priorities and objectives, and adapt and pretest the assessment protocol and data collection tools.

### Site selection

#### National

At the national level, a convenience sample of five sectors were visited to assess the capacities for each of the five IHR(2005) hazards. The sectors included the Ministry of Health Headquarters and two national reference laboratories (Uganda Virus Research Institute and Central Public Health laboratory) for the infectious disease assessment; the Ministry of Agriculture Animal Industry and Fisheries and the Uganda Wild Life Authority for the zoonotic disease assessment; the Radiological Department in Mulago hospital for the radio-nuclear assessment; the National Drug Authority and the Uganda National Bureau of Standards for the food safety assessment; the National Environment Management Authority for the chemical assessment; and Entebbe International Airport, Port Bell and Busia border post for the point of entry assessment. During the sectoral interviews, the hazard focal point officers or other technical officers identified by the sectoral head were selected for administering the adapted assessment tool.

#### District

At the district level, a convenience sample of 13 (16%) districts were selected to assess the core capacity requirements for all the IHR(2005) hazards. In selecting the districts, we considered regional representation from all the four regions of Uganda, districts that are prone to disease outbreaks, and those with major points of entry. The selected districts included Hoima, Busia, Tororo, Moyo, Ntungamo, Bushenyi, Kasese, Arua, Kampala, Mpigi, Wakiso, Bundibugyo, and Kitgum. In each of the selected districts, the assessment was conducted for the district health office, one hospital or a Health Centre IV (HCIV), and three lower level health centres (2 HCIII and 1 HCII). All the facilities were selected randomly using the raffle method in each of the participating districts.

### Capacities measured

The core capacities assessed for each of the five IHR(2005) hazards (infectious, chemical, zoonoses, food safety, radio-nuclear) and points of entry included: national legislation and policy; coordination; surveillance; response; preparedness; risk communication; laboratory; and human resource capacity.

### Data management and analyses

Data entry screens were developed to facilitate data entry using the Epi Info™ [10]. The data were coded prior to entry and following the completion of data entry, the database was cleaned to facilitate the running of frequencies to determine the level of core capacities for each of the five IHR(2005) hazards. Frequency tables and charts were used to summarize the core capacity levels by hazard at national, district and health facility levels. The qualitative analysis by way of strengths, weaknesses, opportunities and threats was used for the interpretation of quantitative data.

## Results

### National legislation for the IHR(2005)

The laws governing surveillance and response for infectious diseases in Uganda were contained in the Public Health (PH) Act (CAP 281). The PH Act provided for cross-border surveillance and the implementation of control measures during a PH emergency. The Act mandated the Minister of Health to declare a disease notifiable. However, the list of notifiable diseases did not include several IHR(2005) notifiable conditions, such as Severe Acute Respiratory Syndrome (SARS), influenza caused by a new sub-type, and smallpox. Similarly, the list of notifiable diseases did not require mandatory notification of chemical and radio-nuclear hazards by public health officers.

The Animal Diseases Act (CAP 38) and the Rabies Act (CAP 44) provided for the notification, surveillance and response to zoonotic events like brucellosis, anthrax, avian influenza and rabies. The Wild Life Act (CAP 200) existed but did not provide for the mandatory notification of zoonoses in wildlife.

The National Environment Act (CAP 153) empowered the local environment committees in districts to report any events or activities which had or were likely to have significant impacts on the environment to the District Environment Officer. There was, however, a need to create mechanisms to ensure that events with public health implications were reported to the District Health Officer and eventually to the IHR NFP.

The Atomic Energy Act of 2008 (CAP 143) provided guidance on the utilization of atomic energy for socio-economic development and ensured that safety standards were upheld through regular inspections of radio-nuclear facilities by the Atomic Energy Council. The legislation, however, did not provide for mandatory notification of accidents, leakage or theft of radioactive sources at radio-nuclear facilities.

The National Food and Drugs Act (CAP 278) provided for the notification of cases of food poisoning to the District Health Officer and the withdrawal of contaminated foods from the shelves.

### Coordination and National Focal Point (NFP) communications

#### IHR coordination

The coordination of emergencies was a mandate of the Office of the Prime Minister (OPM) and was executed by a multi-sectoral Disaster Preparedness and Management committee. There was no disaster management policy and strategic plan addressing the IHR(2005) multi-hazards approach. Public health emergencies were, however, coordinated by a multi-sectoral National Task Force that is chaired by the Ministry of Health. The frequency of the meetings was determined by the presence and nature of a disease outbreak or public health emergency. At the district level, the corresponding structures for coordination of disasters and public health emergencies were the District Disaster Management Committee (DDMC) and the District Epidemic Preparedness and Response committees, respectively. These were functional in 92% (12/13) of the districts, but they only met when there was an emergency.

#### NFP communications and operations

There was a designated IHR NFP that was located in the National Surveillance Unit in the Ministry of Health. Communications from the IHR NFP were done within the IDSR context and were limited to the health sector at the time of this assessment. Operational communication was therefore lacking between the IHR NFP and the other IHR stakeholders located outside the health sector. There were no designated sectoral IHR Focal Points (FPs) to facilitate communications with the IHR NFP in preventing and responding to zoonotic, foodborne, chemical and radio-nuclear hazards. However, the IHR NFP monitored events at the international level through the WHO event information site. Three epidemiologists in the IHR NFP had access to the site and received e-mail alerts of events notified by other countries to the WHO.

The IHR NFP was mandated to provide technical and logistical support to district rapid response teams to conduct the initial health risk assessment and to initiate public health responses during public health emergencies. Further more, the IHR NFP was the national authority responsible for notification of PH emergencies to the WHO. However, systematic use of the decision instrument (Annex 2 of the IHR[2005]) to guide notification by the IHR NFP to the WHO IHR FP was lacking. Only one public health emergency, pandemic influenza (H1N1), had been notified by the IHR NFP to the WHO IHR FP within the 12 months preceding the assessment.

### National advocacy for the IHR(2005)

A sensitisation workshop of key stakeholders on the IHR(2005) had been conducted at the national level by the IHR NFP. However, sub-national sensitisations had not been undertaken and a committee relevant to IHR(2005) implementation had not been established. Similarly, information packages on the IHR(2005) for different target groups and the IHR webpage had not been instituted. At the district level, none of the districts visited had undertaken activities to increase IHR(2005) awareness. Similarly, the IHR information packages were not available in all the districts for distribution to the health facilities.

### Capacities for public health surveillance

#### Detection

Uganda was using the IDSR strategy to conduct surveillance and initiate interventions for the control of infectious and zoonotic diseases in the general population. The national list of priority conditions was limited to the list of IDSR priority conditions, and hence did not include SARS, influenza caused by a new subtype, smallpox, or chemical, radiological, and nuclear hazards.

**Figure 1:**
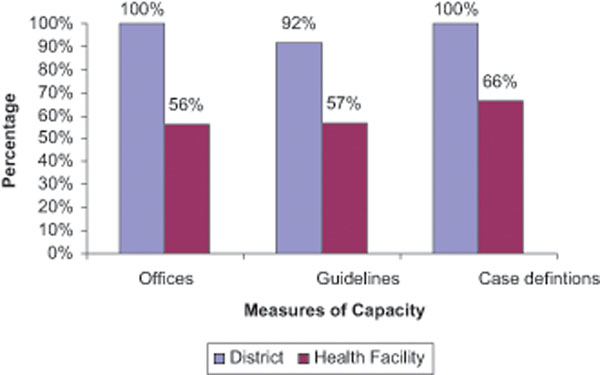
Presence of Public Health Surveillance Offices, Integrated Disease Surveillance and Response Guidelines, and Case Definitions for Infectious Disease Surveillance, Uganda, 2009.

All the districts (13/13) visited had designated public health surveillance offices, most had IDSR technical guidelines (92%, or 12/13), and all (13/13) had case definitions for guidance in case detection of infectious and zoonotic diseases. However, only 57% (35/61) and 66% (40/61) of the health facilities had IDSR technical guidelines and case definitions, respectively, for infectious and zoonotic diseases surveillance (Figure 1). The surveillance guidelines and case definitions on SARS, smallpox, radiological, nuclear and foodborne hazards were lacking at all levels.

#### Reporting

**Figure 2:**
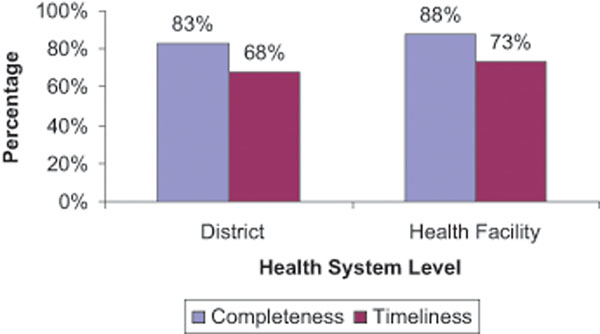
Completeness and Timeliness of Weekly Public Health Surveillance Reporting, District and Health Facility Levels, Uganda, 2009.

Standardized patient registers and report forms were used to collect and report data on infectious and zoonotic events at the health facility level. The forms, however, lacked provision for reporting several IHR priority diseases and hazards. The immediate and weekly reporting of infectious and zoonotic events in humans were largely communicated to the district and national levels by telephone and Short Message Service (SMS) (77%, or 10/13), and to a lesser extent by e-mail and radio call. The National Surveillance Unit, the designated IHR NFP, received the surveillance reports from the districts. The average completeness and timeliness of weekly reporting were assessed for the four weeks preceding the interview. During the period from 31 August to 27 September 2009, the average completeness and timeliness for weekly public health surveillance reporting from the district to the national level was 83% (range 46-100%) and 68% (range 44-100%), respectively. During the same period, the average completeness and timeliness of weekly public health surveillance reporting from the health facility to district level was 88% (range 52-100%) and 73% (range 48-100%), respectively (Figure 2).

#### Data management

The data received at the national level underwent verification and analysis and was thereafter compiled into a weekly epidemiological newsletter for disseminated to all the IDSR stakeholders. All the districts had the capacity to systematically analyze data in terms of person, time and place using either Microsoft Excel, Epi Info™ or paper-based manual analysis. However, only 62% (38/61) of the health facilities had the capacity to systematically conduct basic analysis of epidemiologic data. The majority, 69% (9/13), of the districts reported the availability of a computerized system to analyze epidemiologic data. On the contrary, only 16% (10/61) of the health facilities reported the availability of a computerized system to analyze epidemiologic data.

#### Supervision and feedback

Supervisory visits by the MoH to the districts were few and inconsistent. Only 15% (2/13) of the districts benefited from the supervision of surveillance activities by the MoH in the six months that preceded the assessment. The dissemination of the weekly epidemiologic data was done through weekly newsletters, publication of the data in the newspapers, e-mail, and the monthly IDSR meetings.

#### Cross-border and international surveillance

Cross-border surveillance activities like screening, isolation, quarantine and provision of information were under taken in response to the SARS outbreak in 2003 and pandemic influenza (H1N1) in 2009. The cross-border activities were therefore not routine and barely lasted beyond the prevailing public health threat. However, there were periodic joint cross-border planning and simulation exercises for pandemic influenza under the East African Community (EAC) Secretariat and these could be used to strengthen the IHR(2005) cross-border surveillance capacities as well.

### Capacities for public health response

National and district rapid response teams (RRT) were functional, but their composition did not meet the expertise required for the multi-hazards approach of the IHR(2005). National clinical guidelines for case management of common infections and zoonoses were found in all the districts and in 52% (32/61) of the health facilities. The guidelines, however, lacked procedures for management of chemical and radio-nuclear events. National and district emergency teams, including health care workers at health facility level, had been trained in the management of emergencies due to common infectious and zoonotic hazards. Corresponding training for managing chemical and radio-nuclear hazards had not been undertaken. Projects like Making Medical Injections Safe (MMIS) and other district-based programs were vital in building infection control capacity in the districts, but the country lacked an in-service infection control training program. Medical isolation wards were lacking in all the health facilities visited since they were not included in the standard MoH health facility building plans. Similarly, public health decontamination capabilities for chemical and radio-nuclear hazards were lacking at the national and district levels.

### Capacities for public health preparedness

There were disease-specific national preparedness plans for pandemic influenza, malaria, hepatitis E virus and cholera. The country, however, lacked a comprehensive plan incorporating the all-hazards approach of the IHR(2005). About half (54%) of the districts had preparedness plans, but these also lacked the all-hazards approach of the IHR(2005). The MoH had a roster of experts for supporting infectious hazards response, but it lacked food safety, chemical and radio-nuclear experts.

An assessment of public health emergency needs had not been undertaken at the national level or in 77% (10/13) of the districts visited. Nonetheless, the MoH had a national stockpile that included drugs like Tamiflu, meningitis vaccines and personal protective equipment (PPEs).

### Capacities for risk communication

There was a designated unit in the MoH for risk communication during PH emergencies. The head of the unit was the designated spokesperson for the MoH emergencies. Similarly, all (13/13) of the districts had focal point officers that serve as spokespersons for coordinating risk communication during emergencies. Though the roles, responsibilities and procedures for coordination of risk communication stakeholders were well articulated as part of institutional memory, national guidelines on risk communication were lacking to backup the information.

The MoH had a website which was accessible to the media and public for information dissemination, though it was not regularly updated. Furthermore, the countrywide network of FM radio stations and Village Health Teams offered vital media for disseminating health messages and educating communities. Community messages and materials for the common epidemic diseases (e.g. cholera, meningitis, polio, measles, avian influenza [H5N1] and pandemic influenza [H1N1]) were available at the national level. However, the development of risk communication plans and the mass production of the materials were almost always done during the epidemic season. This therefore usually resulted in risk communication materials not being readily available when outbreaks started. In addition, the scope of the messages fell short of the IHR(2005) all-hazards approach.

### Human resources

An assessment of human resource capacities and corresponding training needs in light of the IHR(2005) multi-hazards approach had not been undertaken. There were at least 17 health training institutions in the country for training public health specialists/epidemiologists, clinical medicine specialists, medical doctors, clinical officers, nurses and other paramedical professions. However, there were no training programs in epidemiology for diploma holders like the district public health surveillance officers and laboratory focal persons, yet they are at the centre of coordinating district surveillance and response activities.

### Laboratory capacities

#### National capacity to deliver laboratory services for all hazards

**Figure 3:**
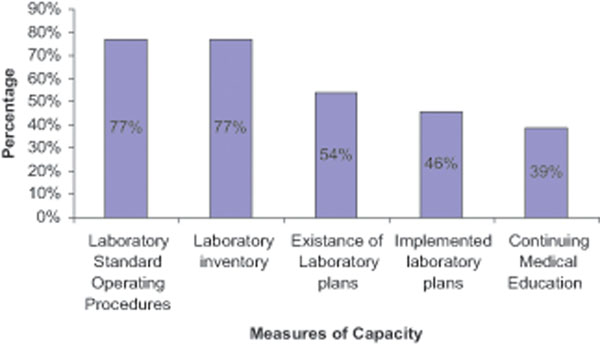
Measures of Capacity for Laboratory Surveillance, District Level, Uganda, 2009 (Districts sampled 16% (13/80)).

The national laboratory policy of 2009 provided for the designation of a national laboratory coordinating office in the MoH and the definition of roles and responsibilities of laboratories at national, regional and district levels with regards to infectious and zoonotic investigations. A five-year strategic plan for strengthening national laboratory services was being drafted by the MoH and health partners. The laboratory standard operating procedures (SOPs) for infectious and zoonotic diseases were available at the national level. Most (77%, or 10/13) of the districts had the laboratory SOPs for infectious and zoonotic diseases. Similarly, most (77%, or 10/13) of the districts had conducted an inventory of laboratory capacity for the various health facility laboratories. However, only 54% (7/13) of the districts had operational plans to strengthen laboratory services and only 46% (6/13) of the districts reported that the plans were being implemented. Only 39% (5/13) of the districts had a plan for continuing professional development of laboratory staff (Figure 3).

**Figure 4:**
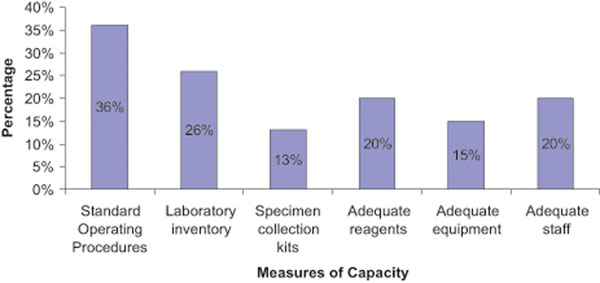
Measures of Laboratory Public Health Surveillance Capacity, Health-facility Level, Uganda, 2010 (Sampled health facilities (n= 61)).

At the health facility level, only 36% (22/61) had laboratory SOPs while only 26% (16/61) had conducted an inventory of their laboratory capacity. A paltry 13% (8/61) of health facilities had adequate specimen collection/ transport materials for routine investigations while 20% (12/61) and 15% (9/61) had adequate lab reagents and adequate lab equipment, respectively. Only 20% (12/61) of the health facilities had adequate staff according to national minimum staffing levels (Figure 4).

#### Confirmation

There was national capacity to confirm priority conditions, namely malaria, Human Immunodeficiency Virus (HIV), tuberculosis (TB), polio, measles, influenza, cholera, Ebola, Marburg, meningococcal meningitis, trypanosomiasis, plague, and yellow fever. In addition, there was a government analytical laboratory with capacity to investigate chemical hazards. However, the country lacked laboratory capacity for investigating radio-nuclear events.

#### Laboratory networking

There was a national laboratory network that functioned through exchange of specimens, data/results, provision of reagents, conducting support supervision and conducting external quality assessments (EQA). The list of designated national reference laboratories was available at the national level and had been disseminated to all levels. The list of international collaborating laboratories for investigating infectious and zoonotic events was available and included the Centers for Disease Control and Prevention (CDC) in Atlanta, Georgia (USA), the National Institute of Communicable Diseases (NICD) in South Africa, and the Kenya Medical Research Institute (KEMRI) in Kenya. However, the list of inter national reference laboratories for radio-nuclear investigations was not available.

#### Specimen collection and transport

There were multiple program-based systems for collecting and transporting specimens to the laboratory. These included the expanded program on immunisation (EPI) system for measles and polio specimens; the influenza sentinel surveillance specimen referral to the National Influenza Centre (NIC); and the postal bus system for sending dry blood spots for early infant diagnosis of HIV. In that regard, 85% (11/13) of districts and 29% (19/65) of health facilities reported having emergency specimen collection kits. However, only 46% (6/13) of the districts and 15% (10/65) of health facilities had transport media while 69% (9/13) of districts and 43% (28/65) of the health facilities had specimen transport carrier boxes.

International air courier services were operating in the country with memoranda of understanding and export permits signed to ship biological specimens. The triple packaging materials were available at the national level and at least three staff working with the national reference laboratories were certified by International Air Transport Association (IATA) for safe shipment of biological specimens. There were, however, no national guide lines to streamline the referral of biological samples to reference international collaborating laboratories.

#### Biosafety and laboratory biosecurity

Despite the absence of a national laboratory biosafety committee, the corresponding guidelines had been developed and disseminated to the districts. The majority of the districts (62%, or 8/13) and a few of the health facilities (22%, or 14/65) reported having copies of the national biosafety SOPs. The country had one high-containment biosafety level (BSL) 3 laboratory and one BSL2+ laboratory, both in the Uganda Virus Research Institute (UVRI).

#### Laboratory quality assurance

Despite the absence of a national laboratory accreditation system, there were four internationally accredited WHO reference laboratories for investigating polio, influenza, plague and tuberculosis. National External Quality Assurance (EQA) schemes using panel testing for HIV, TB and malaria were available. Most of the districts (85%, or 11/13) and about half (49%, or 32/65) of the health facilities reported having a laboratory participating in at least one national EQA scheme. Similarly, the national reference laboratories were participating in international EQA schemes.

#### Laboratory-based surveillance

Standardised tools for collecting and reporting laboratory data were available at the national level. Most of the districts (85%, or 11/13) and 52% (34/65) of the health facilities had the laboratory reporting forms. However, since there was no system for relaying the reports from the district to the national level, the reporting was largely inconsistent, incomplete and not timely. An electronic database existed at the national level and was used for storing and analysing the data.

#### Participation in public health emergencies

The national laboratory services unit reported participating fully in responding to public health emergencies as a member of the National Task Force, national RRT and the IDSR committee.

### Capacities for designated points of entry (PoE)

The country had one international airport (Entebbe), one international port (Port Bell) and several ground crossing points. Uganda had not designated any PoE for the implementation of the IHR(2005) by the time we conducted the assessment. There were no provisions for the application of the following IHR documents at all the points of entry: the International Certificate of Vaccination or prophylaxis; the pertinent health section of the Aircraft General Declaration; the Ship Sanitation Control Certificate/Ship Sanitation Control Exemption Certificate; or the maritime declaration of health. Public health emergency contingency response plans were lacking at all the PoE since there were no permanent public health authorities.

## Discussion

### National legislation for the IHR(2005)

The IHR(2005) mandates each country to fully comply with the obligations therein, but it does not require countries to adopt or revise domestic legislation, provided that they comply with their obligations. That notwithstanding, an adequate legal framework to support and enable all the varied IHR(2005) activities is needed in each country [11]. The revision of national legislation or other instruments should therefore be considered to facilitate full and efficient implementation of the IHR(2005). In this regard, Uganda undertook a process of identifying the relevant national legislation for IHR(2005) implementation. In response to the legal challenges posed in responding to the SARS outbreak in 2003 and the IHR(2005) requirements, countries like Hong Kong revised their infectious disease legislation to facilitate response to public health threats in a timely manner [12]. Niger also amended national legislation to include IHR(2005).

The national legislation in Uganda should be reviewed to facilitate the attainment of the IHR(2005) core capacity requirements.

### Coordination and IHR NFP communications

The presence of a national framework for disaster management is critical for the prevention and management of public health emergencies. The assessment revealed that this framework existed with the Office of the Prime Minister, which had the national mandate for managing disasters, including public health emergencies, in collaboration with the sector- and district-based emergency teams. However, there is need for a disaster management policy and strategic plan that addresses the IHR(2005) multi-hazards approach for managing public health risks and hazards.

Article 4 of the IHR(2005) mandates countries to designate IHR NFPs for coordination of IHR(2005) implementation [5]. Uganda had designated the National Surveillance Unit in the Ministry of Health as the IHR NFP. The scope of activities and operational communication by the IHR NFP was suboptimal, limited to the IDSR strategy for communicable disease control, and excluded PoE. Since partnerships and intersectoral collaboration are essential to IHR(2005) implementation, IHR focal point offices in all the relevant sectors need to be designated to facilitate the establishment of core capacities in line with the IHR(2005) multi-hazards approach.

Additionally, advocacy for the IHR(2005) needs to be prioritized in the country to ensure that all the relevant stakeholders are aware of their obligations and for the mobilisation of the resources required to conduct assessments and to establish and maintain the core capacities for the IHR(2005).

### Capacities for public health surveillance

Article 5 of the IHR(2005) mandates countries to develop and maintain the capacities to detect, assess, notify and report public health events [5]. In Uganda, the IDSR strategy offers a good framework for launching the IHR(2005) implementation given the promising reporting indices. However, the priority diseases list, surveillance guide lines, case definitions and reporting tools have to be updated to incorporate all the IHR(2005) priority diseases and hazards. The revised tools and guidelines should then be disseminated for use by all IHR stakeholders including districts and health facilities since the assessment revealed shortage of surveillance tools and guidelines, especially at the peripheral levels. Cross-border surveillance activities were reactive and only undertaken when there was an ongoing public health risk or hazard. Designation of points of entry for routine implementation of cross-border surveillance activities needs to be undertaken. Article 44 of the IHR(2005) provides for collaboration between countries in developing and maintaining public health capacities [5]. In light of this, the East African Community (EAC) Secretariat initiatives on pandemic influenza preparedness and response should be exploited to strengthen the IHR(2005) core capacities as well.

### Capacities for public health response

Article 13 of the IHR(2005) mandates countries to develop, strengthen and maintain the capacity to respond promptly and effectively to public health risks and public health emergencies of international concern as set out in Annex 1 of the regulations [5]. Following the numerous outbreaks of Ebola in 2000 [13] and 2007 [14], Uganda has accumulated a wealth of experience and expertise for infectious and zoonotic disease response. To this end, there were trained national and district rapid response teams as well as national clinical guidelines for managing infectious and zoonotic disease in the country. However, the membership of the rapid response teams and the content of the outbreak response guidelines needs to be updated to incorporate the IHR(2005) multi-hazards approach.

### Capacities for public health preparedness

Preparedness planning is critical to ensuring that a successful response is mounted in the event of a public health emergency. Uganda had a series of disease specifi c preparedness plans for responding to the recurrent outbreaks of cholera, meningitis, malaria, hepatitis E virus and influenza. However, a comprehensive national plan incorporating the IHR(2005) all-hazards approach has to be developed and replicated at district and peripheral levels. This should go along with building adequate human resources surge capacity and stockpiles of supplies for emergency response to the IHR(2005) hazards.

### Laboratory capacities

Annex 1 of the IHR(2005) requires that countries establish capacities for conducting a comprehensive health risk assessment in response to public health events [5]. Laboratory investigations are central to a comprehensive health risk assessment. Uganda had a laboratory policy that was launched in 2009, and a corresponding laboratory strategic plan was being drafted. It is therefore crucial that the plan provides for the creation of a national biosafety committee, a materials/specimens transfer (referral) policy, a national laboratory accreditation system, and improve the performance of the laboratory information system. Correspondingly though, the national laboratory SOPs were available at the national level, however, critical shortages of laboratory SOPs, reagents, equipment and staffing were observed in the districts. The shortages in laboratory reagents were mainly attributed to poor stock management and hence the need to improve stock management skills and eliminate supply chain bottlenecks.

### Confirmation capacity

The capacities for laboratory investigation of the IHR priority hazards were available except for smallpox and radio-nuclear hazards. In the short term, these gaps could be addressed by identifying a list of international collaborating laboratories that can be readily contacted in the event of a hazard requiring their competencies.

### Other laboratory capacities

Several independent and program-based systems for trans porting polio, measles, influenza and HIV specimens existed in the country. The challenge however, is the need to develop an integrated and cost effective national system to ease the transport of all specimens from the peripheral level to the national level and ensure that the emergency specimen collection kits are available, especially at the peripheral level. National guidelines for material transfer agreements are required to streamline shipping specimens out of the country.

### Capacities at points of entry

The IHR(2005) requires countries to identify and designate PoE for the implementation of measures under Annex 1b [5]. The measures are critical for preventing and controlling international spread of diseases. It is there fore imperative that Uganda designates PoE for the implementation of the IHR(2005) core capacity requirements and builds their capacity for this purpose.

## Conclusions

The assessment highlighted critical gaps to guide the IHR(2005) planning process. The IHR(2005) action plan should therefore be developed to foster improved national and international public health security. This should incorporate the above proposed solutions to the gaps identified with special attention to the recommendations below that are key for smooth implementation of the plan.

## Recommendations

A multisectoral taskforce should be constituted to oversee the amendment of the national laws that are relevant for the IHR(2005). Focal point offices for the IHR should be designated to in all the sectors relevant to the IHR(2005) to facilitate efficient communication with the IHR NFP.

The national priority diseases list, surveillance guidelines, case definitions and reporting tools should be updated to incorporate the IHR(2005). The roster of experts for the national rapid response team and the content of the outbreak response and risk communication guidelines should be updated to incorporate the IHR(2005).

A national preparedness plan that incorporates the IHR(2005) should be developed. The national strategic plan for laboratory services that is being drafted should address the critical gaps identified, including the establishment of a national laboratory accreditation system; the establishment of a national biosafety committee; and the development of guidelines and a national system for specimen referral, both within and outside the country. The PoE for IHR(2005) implementation should be identified and designated.

## Abbreviations

CDC: Centres for Disease Control and Prevention; DDMC: District Disaster Management Committee; EAC: East Africa Community; EQA: External Quality Assurance; FP Focal Point; HC: Health Centre; HIV: Human Immunodefciency Virus; IATA: International Air Transport Association; IDSR: Integrated Disease Surveillance and Response; IHR: International Health Regulations; KEMRI: Kenya Medical Research Institute; MMIS: Making Medical Injections Safe; MOH: Ministry of Health; NFP: National Focal Point; NICD: National Institute of Communicable Diseases; OPM: Office of the Prime Minister; PH: Public Health; PPE: Personal Protective Equipment; PoE: Points of Entry; RRT: Rapid Response Team; SARS: Severe Acute Respiratory Syndrome; SOPs: Standard Operating Procedures; TB: Tuberculosis; USA: United States of America.

## Competing interests

None declared.

## Authors’ contributions

JFW: developed the initial concept, participated in the adaptation of the tools and collection of the data as well as writing of the manuscript and is the corresponding author; CO: contributed to the adaptation of the data collection tools and in the collection of the data and to the writing of the manuscript; IM: technical guidance for the adaptation of the data collection tools as well as writing of the manuscript; NN: developed data entry screens, conducted the data entry and analysis; AK: technical guidance towards the adaptation of the data collection tools, participated in data collection and in writing the manuscript; MN: contributed to the development of the original concept as well as technical guidance for the adaptation and writing of the manuscript; BB: technical guidance on the adaptation of the sections of the tools dealing with laboratory capacities as well as participating in the collection and writing of the manuscript; JJL: technical guidance on the adaptation of the sections of the tools dealing with laboratory capacities as well as participating in the collection and writing of the manuscript; RS: technical guidance for the adaptation of the tools, participated in data collection and writing of the manuscript; JX: technical guidance for the adaptation of the tools, participated in data collection and writing of the manuscript; PG: technical guidance for the adaptation of the tools, participated in data collection and writing of the manuscript; TA: technical guidance on the adaptation of the sections of the tools dealing with laboratory capacities as well as participating in the collection and writing of the manuscript; FDS: technical guidance for the adaptation of the tools, participated in data collection and writing of the manuscript; and SC: technical guidance for the adaptation of the tools, participated in data collection and writing of the manuscript.
